# Understanding and Enhancing Chinese TEFL Teachers’ Motivation for Continuing Professional Development Through the Lens of Self-Determination Theory

**DOI:** 10.3389/fpsyg.2021.768320

**Published:** 2021-11-25

**Authors:** Jie Yang

**Affiliations:** ^1^School of College English Teaching and Research, Henan University, Kaifeng, China; ^2^University International College, Macau University of Science and Technology, Taipa, Macau SAR, China

**Keywords:** motivation, continuing professional development, self-determination theory, TEFL (Teaching English as a Foreign Language) teacher, autonomous motivation

## Abstract

Teachers’ continuing professional development (CPD) is a dynamic process that coordinates teachers’ external behavior and internal states. Most teachers participate in varied forms of CPD activities either voluntarily or contractually. The more motivated teachers are to engage in CPD, the more likely they achieve professional and personal growth. Against this backdrop, the current study, adopting the framework of self-determination theory (SDT), sought to investigate the types and levels of Chinese college TEFL (Teaching English as a Foreign Language, hereafter TEFL) teachers’ motivation toward CPD. The questionnaire survey was administered to 402 academics who taught the English language in diverse universities in China. The quantitative analysis showed that teachers exhibited stronger identified regulation and intrinsic motivation than external regulation. Specifically, the exploratory factor analysis identified five motivational orientations: inner-directed academic improvement and cognitive interest, academic self-fulfillment and obligation, academic and social responsibility, social recognition and promotion, lacking the intention for CPD, which corresponded to the SDT motivation continuum. To triangulate, further interviews were conducted with 12 TEFL teachers. The findings of the qualitative analysis—the thematic analysis through Nvivo (Version 12) on the open-ended question and the interviews, revealed that teachers’ struggles in CPD were mainly concerned with the school-related, CPD-related, and teacher-related problems. Moreover, a big gap between teachers’ needs and contextual support was found to be bridged. Based on the findings, this study proposed that social conditions should support teachers’ basic psychological needs in order to sustain and enhance TEFL teachers’ motivation for CPD.

## Introduction

Teachers’ continuing professional development (CPD) is a continuous and lifelong process whereby teachers could manage their own personal growth and professional learning. Broadly defined, CPD is “a term used to describe all the activities in which teachers engage during the course of a career which are designed to enhance their work” (Day and Sachs, 2004, p. 3). Although the types of CPD, such as courses, workshops, education conferences or seminars, qualification programs, etc., vary widely in their content and format, most of which share the common purpose of maintaining and improving education quality. As a contributing factor to the improvement of student learning and teaching practice (Guskey, 2003; Caena, 2011; OECD, 2014; Kennedy, 2016; Anđić, 2020), CPD has been attached great importance in nourishing teacher quality in the national and international context. In many countries, relevant policies have been enacted in order to trigger teachers’ motivation for participating in various CPD programs. Motivation is the reason “why people decide to do what they do, how long they are willing to sustain the activity and how hard they are going to pursue it” (Dörnyei, 2001a, p. 7).

Previous research has documented that when intrinsically motivated, the person can achieve and sustain more accomplishment as well as experiencing a great level of well-being (Ryan and Deci, 2000a,b; Pishghadam et al., 2021), and motivated teachers are more likely to support students’ autonomy and enhance the intrinsic motivation amongst students (Pelletier et al., 2002). In the domain of professional development, this factor, that is, “what motivated teachers to engage in professional development” (Guskey, 2002, p. 382), also makes a big difference to the effectiveness of those professional development programs. This is why many researchers, such as Boyd et al. (2003) and Tittle (2006) echoed the call by emphasizing the importance of attracting teachers and sustaining their involvement in professional development through motivation studies. It is said teachers’ motivation determined what would happen after their participation in CPD (Osman and Warner, 2020), and those teachers with a strong autonomous motivation are more likely to integrate new practices into their teaching (Wal et al., 2014; Gorozidis and Papaioannou, 2016). The findings of a plethora of studies have attested to the benefits and desirable consequences autonomous motivation or intrinsic motivation brought to formal education (Froiland and Worrell, 2016; Power and Goodnough, 2018; Ryan and Deci, 2020). However, fostering such motives is not an easy task. From the perspective of SDT theory (an organismic-dialectic framework of human motivation and development), it only occurs under conditions that support individuals’ psychological needs of feeling competent, related, and autonomous within their actions (Deci and Ryan, 2008a,b).

In other words, to achieve teachers’ optimal learning in CPD and optimal implementation after CPD, teachers need to be strongly motivated in terms of the more autonomous motivational regulations as proposed by SDT theory. This is especially important for TEFL teachers in the Chinese context, as they are undergoing the transition from teaching EGP (English for General Purpose) to ESP (English for Special Purpose)/EAP (English for Academic Purpose) (Cai, 2017). This initiative in college English language teaching has presented teachers with challenges for their professional learning. They are likely to face various sorts of obstacles as they adapt to the change. In addition, their motivation may be distinguished from that of other social and cultural situations, showing the structure and characteristics of a collective culture in which individuals tend to internalize the demands of people they feel attached to Bao and Lam (2008). Despite this cultural difference, another factor concerning the importance of investigating Chinese teachers’ CPD motivation is that the extant research in the Chinese context stands in stark contrast to that of western societies where most studies relevant to CPD motivation have been carried out (Xu and Cai, 2013; Zhang et al., 2021).

Therefore, being exploratory in nature, the current study intends to provide insights into the factors that explain Chinese TEFL teachers’ motivation for CPD. The results, discussed in light of SDT theory, could add to our understanding of the professional development of language teachers in a particular social and cultural background, provide more practical applications of the motivation theory in a significant component of education — teacher education and facilitate teachers in fostering authentic self-determination in themselves as well.

### Literature Review

As far as teaching and teacher education are concerned, motivation “determines what attracts individuals to teaching, how long they remain in their initial teacher education courses and subsequently the teaching profession, and the extent to which they engage with their courses and the profession” (Sinclair, 2008, p. 80). Generally speaking, as a broad and complex concept, teacher motivation is usually defined in accordance with the research purposes. It could be subdivided into more specific dimensions from various view points, for example, teachers’ motivation for teaching, teachers’ motivation for research and teachers’ motivation for professional development, to name but a few. Such subdivision presents content for the understanding of teacher motivation. Apart from the perspective of the content of teacher motivation, the existing literature also tends to delve into the field in terms of pre-service teacher motivation and in-service teacher motivation (Han and Yin, 2016). In this sense, the current study concentrates on in-service teachers’ motivation with the content of continuous professional development.

### Theoretical Perspectives of Teacher Motivation

In the domain of teacher education studies, the theoretical frameworks mainly generated from social-cognitive theories of motivation, for example, expectancy-value theory, achievement goal theory and self-determination theory. Expectancy-value theory considers that individuals’ motivation to complete tasks is determined by their expectation of how well they will do on the task and the extent to which they value the activity (Wigfield and Eccles, 2000). One of the important researches adopting this framework was done by Richardson and Watt (2006). They conducted a large-scale study to determine the strength of influence for a range of motivations from pre-service teachers across three universities in Australia. As for achievement goal theory, theorists argue that the choice of individuals’ achievement goals depends on their abilities and characteristics (Senko et al., 2011). These two classical theories have been applied in the field of teacher motivation for the understanding of teachers’, especially pre-service teachers’ psychological factors influencing their decision in choosing the teaching career as well as their dedication to the teaching career.

In addition to these two motivation theories, another classical and significant theory that has been extensively employed to interpret teacher motivation studies is self-determination theory (SDT). SDT theorizes human motivation into three main categories: intrinsic motivation, extrinsic motivation and amotivation. Intrinsic motivation expresses and represents the positive potential of human nature, pertaining to activities done for their inherent interest and enjoyment, or in other words, for their own sake. It reflects a state of a high degree of self-determination. Extrinsic motivation, in contrary to intrinsic motivation, concerns behavior done for external incentives or rewards, such as economic struggle, fame pursuit, physical attraction. It can be further subdivided into four major types, namely: external regulation (concerns actions taken to satisfy external contingencies), introjected regulation (indicates individual’s behavior is regulated by self-imposed pressure in order to avoid anxiety and guilt or to improve self-esteem), identified regulation (individual’s recognition and endorsement of the value of an action), integrated regulation (refers to the individual’s full assimilation of the identified regulation). Amotivation implies an individual’s state of lacking the intention to act.

These types of motivations have been identified and confirmed in various empirical studies (Han and Yin, 2016; Howard et al., 2017; Roth et al., 2007). Furthermore, from the perspective of SDT, the aforementioned motivational types are placed on a continuum of self-determination, one end of which reflects the most highly self-determined type of motivation, namely intrinsic motivation, whilst the other end, that is, amotivation, signifies the least self-determined type. According to the degree of the internalization of one’s behavior regulation, SDT also posits a controlled-to-autonomous continuum to describe the degree to which an external regulation has been internalized (Gagne and Deci, 2005). Along this continuum, the external regulation is a prototype of controlled motivation while intrinsic motivation, marked by an individual’s participation in activities with the sense of volition and self-endorsement, is considered a typical example of autonomous motivation. Other types of regulation, introjected, identified, and integrated ones, vary in the degree to which they are autonomous versus controlled (Deci and Ryan, 2008a). The more the individual values an activity, integrates and internalizes its importance, the more likely he is to be autonomously motivated. The benefits of intrinsic motivation or autonomous motivation are obvious within formal education (Ryan and Deci, 2020). Various empirical studies have pointed to the critical role they played in school achievement. For example, the meta-analysis of Taylor et al. (2014) showed intrinsic motivation was consistently related to higher performance; Froiland and Worrell (2016), through their study in a diverse high school, came to the conclusion that intrinsic motivation could predict student engagement, which in turn predicted students’ higher achievement. Additionally, the study of Hein et al. (2012) also confirmed the positive relationship between teachers’ autonomous motivation and their productive (student-centered) teaching style.

However, individuals’ inherent or autonomous motivational propensities for learning and growing are not seen as automatic. Instead, they require supportive condition to become robust. Specifically, SDT distinguishes people’s three innate psychological needs: competence, autonomy, and relatedness. “Competence” refers to individuals’ feeling of mastery, a sense that one can succeed or grow; “autonomy” concerns individuals’ sense of initiative and ownership in their own actions; “relatedness” is associated with individuals’ sense of belonging and connection (Ryan and Deci, 2020). These fundamental psychological needs are the key to persons’ healthy development. According to SDT, individuals’ perceptions of context as supportive of autonomy or controlling make a big difference to the impact of external contingencies on their intrinsic motivation and self-determination (Han and Yin, 2016). In other words, when all three needs are satisfied, the individual could perceive his behavior is internally initiated, self-controlled and regulated, thus achieving better psychological well-being (Ryan and Deci, 2000b). This kind of well-being is closely related to positive psychology, leading to people’s actualization of potentials and social development (Dewaele et al., 2019; MacIntyre et al., 2019; Wang et al., 2021).

As a broad framework to understand factors that facilitate or undermine intrinsic motivation, autonomous extrinsic motivation, and psychological wellness, all issues of direct relevance to educational settings (Ryan and Deci, 2020), SDT provides an appropriate angle for the current study exploring teachers’ motivation for professional development in a particular context.

### Research of Teachers’ Continuing Professional Development

The rationale for prioritizing teacher quality in the educational system has stemmed from the consensus that “enhancing teacher quality is key to improving the quality of an educational system more generally” (Borg, 2018, p. 1). As the priority at various educational levels at home and abroad, the issue of teacher quality allows for and requires academics to continually participate in a variety of professional development activities. Attending CPD is considered a quality showing teachers the right way in their careers (Derakhshan et al., 2020a). These in-service learning experiences are considered to be able to facilitate teachers’ personal and professional identity and growth, driving positive changes in them and making them more autonomous and successful (Derakhshan et al., 2020b). However, this underlying assumption of teachers’ CPD that it could empower academics with content pedagogical knowledge and motivate them to transform new ideas into their instructional practice with the result of promoting student learning outcomes has been questioned by many researchers. The reviews of CPD studies have shown the mixed evidence of the effectiveness of CPD programs (Lawless and Pellegrino, 2007; Guskyey and Yoon, 2009; Gegenfurtner, 2011; Opfer and Pedder, 2011). The main cause leading to the inconsistency of the vision of CPD lies in the idiosyncrasies of individual academics and the different educational settings in which they work (Osman and Warner, 2020). Teachers differ in their implementation of the objectives of CPD programs due to both internal and external factors (Kennedy, 2016). One of the crucial internal factors inducing the failure of many programs is the neglect of teachers’ motivation, which was suggested in many studies (Guskey, 1986; Opfer and Pedder, 2011; Kennedy, 2016). For instance, Ng (2010) argued that “little is known about what factors can motivate teachers to engage in professional learning in a meaningful way” (p. 397). This view was echoed by McMillan et al. (2016), who also emphasized the significance of teachers’ motivation in CPD. In fact, since Guskey appealed for more attention to the issue of teacher’s motivation in professional learning, a plethora of studies have been providing more clarity on the role teachers’ motivation plays in the realm of CPD. According to Wal et al. (2014), school teachers with more autonomous profiles got more involved in professional development programs as they valued and enjoyed the activities *per se* more and they were conscious of the significance of those programs. Likewise, the conclusion of Gorozidis and Papaioannou’s (2016) study demonstrated the positive association between autonomous motivation and teachers’ intentions to engage in CPD. Similarly, from the angle of intrinsic and extrinsic motivation, Liu et al. (2019) stated that stable intrinsic motivation can work as the ideal support for teachers’ high-level development and it can come into being when teachers integrate personal and professional development into intrinsic values.

### Self-Determination Theory and Teacher Motivation Research

Self-determination theory has been one of the motivational theories applied to teacher motivation research and it has been primarily used to address the relationships between teacher motivation and student motivation (Deci et al., 1982; Han and Yin, 2016). Deci et al. (1982) suggested that the more teachers were pressured by superiors, the more they were likely to be controlling on students. This finding was supported by the research done by Pelletier et al. (2002), who used structural equation modeling to illustrate the relationships between teacher motivation and student motivation. Their research results showed that teachers perceiving more constraints at different levels of educational setting along with their perceptions of students’ low self-determination, could lead to their less self-determined teaching and more controlling on students. In other words, students’ intrinsic motivation and determination were influenced significantly by the extent to which teachers were autonomous-supportive or controlling (Radel et al., 2010).

As research on SDT has received increasing attention, several studies disputing the structure (Chemolli and Gagné, 2014), or confirming the ordering (Howard et al., 2017), as well as providing a nuanced interpretation of the issue of both self-determination and specificity of motives (Litalien et al., 2017; Howard et al., 2018), have provided the empirical evidence for the deepened apprehension of SDT in recent years. Apart from research examining the unique properties of intrinsic and extrinsic motivation, there is also a growing interest in the predictability of these motives across varied cultural contexts and educational levels. Jang et al. (2009) showed that autonomy was predictive of students’ satisfaction on their learning experience in the South Korean context. Oga-Baldwin et al. (2017) found that Japanese students’ language learning engagement could be enhanced by promoting intrinsic motivation and autonomy.

In addition to the research concerning student motivation, and the relationship between teacher motivation and student motivation, SDT has also been employed to analyze teachers’ competence in differentiating motivational types in terms of autonomy (Roth et al., 2007). The results provided supportive evidence for the application of SDT framework in teacher education studies. There were positive linkages between teachers’ motivation and teacher learning outcomes (Gagné et al., 2010).

Among the studies on teacher motivation within specific disciplines, language teacher motivation has received increasing attention in the past decades. Pennington (1995) approached the topic through her studies concerning ESL (English as a Second Language) teachers’ job satisfaction. Besides, the influencing factors motivating or demotivating language teachers have become a topic extensively explored on a wide scale. Factors such as quality work, teachers’ enthusiasm and commitment, students’ level of learning, etc., have been identified as determinants in correlation with teachers’ motivation (Praver and Oga-Baldwin, 2008; Sugino, 2010; Erkaya, 2012). In sum, empirical studies have pointed to language teachers’ dominance of intrinsic motivation over extrinsic motivation (Kassabgy et al., 2001; Erkaya, 2012).

As can be seen from the literature, teachers’ or language teachers’ intrinsic or autonomous motivation, matters significantly, and it is possible to transform extrinsic motivation into intrinsic one. However, despite the increasing attention on the aforementioned aspects, language teachers’ motivation for continuing training still requires more efforts. As Han and Yin (2016) in their review article of teacher motivation pointed out contextual issues remains an important aspect underexplored. From their research, discipline and social cultural context are two significant considerations for the future teacher motivation research.

The previous findings offer inspiration to researchers in the domain of teacher education in the Chinese context. As one of the external factors influencing the effectiveness of professional development, the cultural context has been a variable explored in the existing literature. For example, Liu and Onwuegbuzie (2014) adopted a concurrent equal-status design to delve into the factors motivating Chinese teachers as opposed to the findings presented in the international literature. Ren and Xu (2005) designed a questionnaire to investigate school teachers’ motivation for taking the Master’s degree program, and the results unfolded mainly four motivations: career development, external influences, professional growth, and competitive advantage. By investigating the scholarly visiting motivation of TEFL teachers in Chinese higher institutes, Liu and Kou (2014) extracted seven factors in relation to their education participation intention. Moreover, Xie et al. (2014) explored Chinese university teachers’ research motivation under the guidance of intrinsic and extrinsic motivation.

The reviewed empirical literature above uncovers the fact that motivation makes a big difference to teachers’ professional development and a strong intrinsic or autonomous motive can greatly promote teachers’ growth in their career journey. However, we can also notice there appear to be many unexplored matters about teachers’ motivation in the Chinese context as the CPD becomes increasingly critical in the process of China’s initiative to build “first-class” universities. One important issue that remained to be addressed is TEFL teachers’ motivation for CPD. Currently, few studies have scrutinized the truth of TEFL teachers’ motivation for CPD (Yu, 2019), and their needs for development are still poorly understood.

As a major site of English language teaching activity worldwide (Borg and Liu, 2013), China has initiated the English language teaching reform among most higher education institutes, which aims to promote English for Special Purpose (ESP), especially English for Academic Purpose (EAP)-oriented curriculum transformation (Zhang and Deng, 2020). The change of the focus from traditional general English teaching to ESP and EAP teaching has posed a challenge for TEFL teachers’ professional learning. Moreover, the duration of COVID-19 pandemic also has thwarted teachers’ attempts in engaging in and benefiting from especially the traditional professional development programs. Against such a backdrop, the focus on motivation could be a proper research angle for investigating teachers’ psychological needs and values, furthering the understanding of the effectiveness of CPD.

This research thus seeks to expand upon the previous findings, concentrating on the motivation of TEFL teachers in various higher institutes in the Chinese context in terms of why they engage in CPD and how to sustain their motivation for CPD. To tackle these issues, the following research questions are addressed:

(1) What are the motivations of Chinese TEFL teachers to undertake in-service professional development training?
(2) To what extent are Chinese TEFL teachers intrinsically and extrinsically motivated to continue their professional development activities? Are there any differences among teachers of different demographic background?
(3) What problems arise in sustaining Chinese TEFL teachers’ motivation for continuing professional development?

## Materials and Methods

### Participants

The researcher surveyed 402 teachers who taught English language and related courses in various higher education institutes across China. They were from universities or colleges of different affiliations and geographical locations, with, 72 (17.9%) male teachers and 330 (82.1%) female teachers participating in varied CPD programs in the past 5 years. The distribution and description of the survey sample are listed in Table 1.

**TABLE 1:** Sample description and distribution.

Demographic variables	Categorical groups	*N*	%
Gender	Male	72	17.9
	Female	330	82.1
Education background	Bachelor	52	12.9
	Master	301	74.9
	Doctor	49	12.2
Age	20–30	51	12.7
	30–39	178	44.3
	40–49	125	31.1
	50–59	41	10.2
	60 or above	7	1.7
Years in teaching	Less than 5	90	22.4
	5–10	68	16.9
	11–20	148	36.8
	Above 20	96	23.9
Title	Assistant	68	16.9
	Lecturer	189	47.1
	Associate professor	130	32.3
	Professor	15	3.7
Affiliation	Ordinary university	222	55.2
	Provincial key university	71	17.7
	Project 211 university	60	14.9
	Project 985 university	9	2.2
	Higher vocational colleges	26	6.5
	Others	14	3.5

It should be explained that there are generally two types of university departments recruiting English teachers in the Chinese context, one of which is called the College English Teaching Department (the research subjects of the current study) and the other one is usually termed English Language and Literature Department. The former’s mission is to teach general English language skills to students of non-English majors (These English courses usually are part of the compulsory general education curriculum), whilst the latter concentrates on offering English-major students courses like English linguistics, translation or literature.

The follow-up interviews, focusing on how to sustain and enhance teachers’ motivation, were conducted with 12 respondents from nine universities who were randomly selected from those participants who left their contact information and showed interest to be interviewed. Each interview session took approximately 20 minutes. In order to obtain a more accurate and reliable dataset, the interviews were held in participants’ mother tongue. The participants’ answers were then translated into English for further content analysis.

### Research Instruments

The mixed-methods research design was adopted in this study. Therefore, questionnaires and interviews were employed as major research instruments. The questionnaire employed to gather data contained three parts. Part 1 elicited the survey respondents’ demographic information as shown in Table 1. Part 2 was composed of one multiple-choice item describing the information with regard to the CPD programs participants have taken in the last 5 years and a 34-item scale investigating the types of teachers’ CPD motivation. These 34 items were measured on a five-point Likert scale that ranged from 1 = strongly disagree to 5 = strongly agree. Part 3 comprised one open-ended question (item 35), asking respondents to list the problems or challenges they encountered in the CPD programs. The survey items measuring TEFL teachers’ CPD motivation were largely based on the questionnaires developed by Peng and Gao (2019) and Liu et al. (2020), with the supplement of some items from Boshier’s (1991) research. Minor modifications were made to better suit the current research purposes. Additionally, the survey was written in Chinese and answered anonymously.

The interviews of the 12 TEFL teachers were supportive in further data triangulation. The interview questions were mainly designed on the basis of the answers of open-ended item exploring the problems in TEFL teachers’ CPD experiences. Through the interviews, the study managed to verify the interviewees’ motivation for CPD, acquire insight about the difficulties they had and their suggestions in sustaining and enhancing the CPD motivation.

It should be mentioned that the current study exploring language teachers’ motivation for CPD was under the guidance of SDT framework. As a classical motivation theory, SDT has generated many instruments developed by researchers (Roth et al., 2007; Fernet et al., 2008). However, the pilot study of this research showed that these standardized questionnaires need to be adapted for taking into consideration the particular Chinese context. For example, the first step of the pilot study, that is, gathering Chinese TEFL teachers’ CPD motivation via open-ended questions (e.g., what motivated you to take part in CPD activities?), demonstrated a factor frequently mentioned by participants—to supplement a narrow previous education (Q 28). This was due to the policy change concerning TEFL teachers’ recruitment.

To be specific, in the Chinese context, the threshold for entering the College English Teaching Department was the holding of a master’s degree in the English language-relevant domains several years ago. However, with the increasingly fierce competition in the talent market and the launching of the initiative to build “Double First-Class Scheme” in China (to build “first-class” universities and “first-class” academic disciplines) (State Council, 2015), this threshold has been raised, and a doctoral degree is required. Meanwhile, the requirement for research and scholarly publishing has become a more significant indicator in TEFL teachers’ performance assessment and career advancement. The change has also been reflected in the questionnaire survey in which 68 (16.9%) teachers selected “Ph.D. degree program” as one of the CPD projects they have participated in and many teachers complained about the excessive attention on research and publication related CPD programs in response to their perception of the current CPD activities. Therefore, it was possible that teachers were motivated and challenged by this transform in the higher educational setting. Additionally, there were some other factors of Chinese-context characteristics that need to be noticed in the pilot study, for instance, to gain recognition from colleagues and superior leaders (Q8, Q32). As a result, the current research chose to adapt and develop a questionnaire taking both SDT framework and Chinese context into consideration.

### Data Collection

Before formal questionnaire collection, the pilot study comprising two steps was conducted. First, based on the results of interviews with 15 Chinese TEFL teachers and the used standardized questionnaires (Roth et al., 2007; Fernet et al., 2008; Peng and Gao, 2019), a questionnaire concerning teachers’ motivation for CPD was designed and distributed to 60 TEFL teachers at different higher educational institutes. The second step then was the analysis and summary of the answers of participants. After pilot study, formal questionnaires were issued and collected.

In particular, the data collection commenced in May 2021 and lasted for about 4 months. During this period, the survey was administered via a Chinese online questionnaire system named Wenjuanxing^1^, which is popular and widely used in China’s mainland. To promote participation, the questionnaire link was also specifically sent to many a Wechat (a Chinese multi-purpose messaging, social media, and mobile payment app) group of TEFL teachers. The follow-up interviews were conducted with 12 TEFL teachers who were willing to share their CPD experiences and comments. These teachers were purposively selected and they are three males and nine females (two professors, five associated professors, and five lecturers). As the semi-structured interviews were conducted to delve into issues in sustaining and enhancing teachers’ motivation for CPD, questions were asked to figure out their specific motivation for CPD, the problems they encountered in their CPD experiences, and their comments and suggestions on the current CPD programs. Besides, all the 12 interviews were carried out online in Mandarin (the native language of the interviewees) due to the COVID-19 pandemic. Each interview lasted for about 20 minutes and was audio-recorded for later encoding. The transcriptions of the audios were sent to the interviewees for a double-check of accuracy. Ethics approval was granted by the interviewees who remained confidential and informed consent was gained from respondents before their participation in the study. None of the interviewees had a conflict of interest with the researcher.

### Data Analysis

First, the data collected from the questionnaire were used to address the first and second research questions. As is mentioned previously, the research questionnaire for investigating Chinese TEFL teachers’ CPD motivation was adapted and designed on the basis of the existing questionnaires, taking the specific research context and purpose into consideration. Remained a theme to be explored and understood, TEFL teachers’ motivation for CPD was vague and uncertain, so exploratory factor analysis (EFA) was necessary to identify the observed and potential variables, their factor structure as well. Specifically, principal components analysis (PCA) with Promax rotation was adopted to examine the factor structure of the 34 items in the EFA analysis. Furthermore, *t*-test and one-way ANOVA were conducted to identify the differences in the level of teachers’ CPD motivation in terms of their demographic information (such as gender, years in teaching, etc.).

Second, qualitative content analysis was applied to address the third research question, namely, “how to sustain and enhance teachers’ CPD motivation.” It is widely accepted that content analysis can add interpretive depth to the research analysis, enabling researchers to explore deeper in their efforts (Dörnyei and Taguchi, 2011). Therefore, this method was carried out to analyze both the responses to the open-ended question (item 35) and the transcription of the interviews from 12 TEFL teachers. Content analysis can be implemented either deductively or inductively. Deductive method refers to researchers’ drawing on a theoretical model while analyzing qualitative data whereas in the inductive approach, researchers dealing with data without any prior model (Berg, 2002). In this inquiry, the inductive method was opted to analyze the participants’ responses. Specifically, the codification process was done through a qualitative analysis software — Nvivo (Version 12). To improve the credibility of the codification, the author invited a researcher who is skilled in coding the content to participate in all the stages of data analysis. The coding process mainly comprised three steps: open coding, creating categories, and abstraction. In the first step, the data were read multiple times and 54 codes for the difficulties and challenges teachers encountered were generated. The second step, termed “creating categories,” was to compare and group the open codes generated in the first step under related themes. This step generated 24 relevant subthemes. Next, in the third step — the abstraction, 24 subthemes were categorized into three major themes, namely, school-related problems, CPD-related problems, teacher-related problems. The inter-coder agreement index through Cohen’s Kappa coefficient turned out to be 0.85. Furthermore, to carry out “member checking” (Lincoln and Guba, 1985), the data of the interview codification were also given to the interviewees to obtain their feedback on the clarity and accuracy of the codification. The interviewees approved the generated themes and codes in general. Finally, for the confirmability (Lincoln and Guba, 1985; Derakhshan et al., 2021) of the research, the whole data together with the generated codes and themes were provided to an outside researcher for audit trial. The areas of disagreement were resolved by means of discussion and compromise.

## Results and Discussion

### Dimensions of TEFL Teachers’ Continuing Professional Development Motivation

The results of reliability statistics indicated that the questionnaire for teachers’ CPD motivation was inherently consistent with Cronbach’s Alpha ratio reaching 0.906. The validity test showed the Kaiser–Meyer–Olkin (KMO) value attained 0.907 (χ^2^ = 7046.324, df = 561, *p* = 0.000), which pointed to a suitable sampling for EFA analysis. To respond to the first question, multiple methods were used to determine the factor structure of the 34 items (e.g., eigenvalue-greater-than-one, parallel analysis). And after the deletion of eight items, eventually, five factors were extracted, explaining 59.498% of all variances cumulatively (see Table 2). All the factor loadings were above 0.40 and all the eigenvalues of these factors were greater than 1. In addition, the reliability coefficients of the five factors range from 0.719 to 0.870, indicating good internal consistency reliability indices.

**TABLE 2:** Results of EFA for TEFL teachers’ CPD motivation.

No. item		Factor				
		1. Social recognition and promotion	2. Inner-directed academic improvement and cognitive interest	3. Lacking the intention for CPD	4. Academic and social responsibility	5. Academic self-fulfillment and obligation
33	I’m engaged in CPD programs to improve my social relationships.	**0.882**	−0.209	0.094	0.062	0.065
34	I participate in CPD programs to maintain and improve my social status.	**0.862**	−0.166	0.024	0.071	0.058
32	I participate in CPD programs to be accepted by others.	**0.826**	−0.243	0.208	0.023	0.099
8	I’m engaged in CPD programs to gain recognition from my colleagues.	**0.761**	0.115	−0.114	−0.089	0.157
7	I’m engaged in CPD programs to increase income.	**0.718**	0.288	−0.029	−0.203	0.028
14	14. I’m engaged in CPD programs to catch up with others.	**0.531**	0.057	−0.028	0.189	−0.077
29	I participated in CPD programs to gain promotion.	**0.518**	0.254	−0.101	0.092	−0.260
20	I’ m engaged in CPD programs to realize my self-worth.	0.121	**0.795**	−0.039	−0.136	0.018
18	I’m engaged in CPD programs to satisfy my inquiring mind.	−0.159	**0.787**	0.097	0.071	0.110
19	I’m engaged in CPD programs for the pursuit of high-level academic papers.	0.256	**0.778**	−0.002	−0.175	−0.183
23	The main factor inspiring me to engage in CPD is that I want to improve my academic level and knowledge.	−0.158	**0.674**	−0.050	0.189	0.025
15	I very much like the feeling of constantly exploring and researching.	−0.163	**0.643**	0.042	0.154	0.104
16	I’m engaged in CPD programs to improve my critical thinking ability.	−0.039	**0.582**	0.000	0.116	0.247
22	Being able to contribute to relevant academic field is the main factor that motivates me to engage in CPD.	0.058	**0.530**	0.070	0.255	−0.029
26	I do not see the relevance of going to the CPD programs.	0.006	0.021	**0.892**	−0.056	−0.046
31	I do not know the reasons of participating in CPD.	0.074	−0.068	**0.845**	0.018	0.045
21	I do not understand the purpose of going to the CPD programs.	−0.038	0.142	**0.787**	0.130	−0.062
17	I participate in CPD programs because my school supports them.	−0.054	0.016	0.052	**0.754**	0.042
25	I participate in CPD programs to meet the requirements of performance evaluation in my school.	0.216	0.074	0.176	**0.504**	−0.228
24	I’m engaged in CPD programs to fulfill a kind of social responsibility.	0.064	0.246	0.169	**0.495**	0.121
27	I’m engaged in CPD programs to keep up with competition.	0.374	0.059	−0.160	**0.430**	−0.092
28	I’ m engaged in CPD programs to supplement a narrow previous education.	0.238	0.146	−0.218	**0.423**	0.058
2	To seek knowledge for its own sake is the main driving force for me to participate in CPD.	0.036	0.219	0.072	−0.155	**0.752**
1	To better guide my teaching is the main driving force for me to participate in CPD.	0.025	−0.032	−0.118	0.135	**0.730**
3	I’m engaged in CPD programs for my future academic development.	0.091	0.397	0.043	−0.108	**0.537**
4	It is teachers’ duty to participate in CPD programs.	0.011	−0.124	−0.074	0.476	**0.517**
Cronbach’s α		**0.870**	**0.849**	**0.816**	**0.723**	**0.719**

*Factor loadings above 0.40 are shown in bold.*

Factor 1 — Social recognition and promotion. This factor reflects that teachers’ motivation for CPD was influenced by external factors and stimuli. It mainly encompasses two aspects: one is related to the respondents’ desire to strive for social status, improve social relations and gain social recognition (Q33, Q34, Q32, Q8). As members of society, TEFL teachers are undoubtedly influenced by the social environment. They are often energized into action with the intention of gaining a desired external consequence from their work. These orientations for social rewards were supported by a large number of empirical studies (Boshier, 1991; Howard et al., 2021).

The other aspect attests to teachers’ intention for a salary increase, job promotion, and competition needs (Q7, Q14, Q29). Both orientations are behavioral tendencies induced by external causes. From the perspective of SDT theory, this motivation mainly belongs to external regulation, referring to doing something for a separable outcome. Although SDT theory recognizes the short-term efficacy of external regulation, it also portrays it as a form of motivation of low quality that is often undermining individuals’ self-determined motives (Deci et al., 1999; Ryan and Deci, 2020). Overall, this factor is typically a form of external motivation that is opposite to internal motivation. In this case of CPD, teachers’ behavior is non-autonomous and controlled by external contingencies.

Factor 2 — Cognitive interest and inner-directed academic improvement. It points to teachers’ desires to obtain more knowledge, expand their horizons, and contribute to their own academic fields. Items 18, 23, 15, 16 are a reflection of teachers’ keen and genuine interest in satisfying their own needs for knowledge in the field. Items 20, 19, 22 are related to teachers’ intentions to improve themselves and contribute to their research realm academically. This motivation is inner-directed, concentrating on gaining the psychological reward rather than physical. Speaking in general terms, it is the intrinsic motivation, indicating that “people performing a behavior for its own sake in order to experience pleasure and satisfaction …” (Dörnyei, 2001b, p. 47). Item 19 “the pursuit of high-level academic papers,” shares similarities with many prior studies (Bai and Hudson, 2011; Borg and Liu, 2013; Song, 2017; Gao and Zheng, 2018), which highlighted the prioritization of publication for English language teachers in the Chinese universities. However, rather than just “publishing for career promotion,” Peng and Gao’s (2019) study argued that many teachers in the field doing research for a better guiding and informing of their pedagogical practices, which was in consistency with some studies (see Bai and Hudson, 2011; Bai, 2018). Therefore, teachers’ pursuit of high-quality papers was not necessarily the result of external stimulation. Instead, it could stem from their own need for “research for teaching.” Indeed, according to the content analysis of this research interview, the suggestions of some teachers just revealed a fact that there was a strong need for pedagogical practices-related CPD programs. In sum, TEFL teachers’ pursuit of academic knowledge and academic papers reflected their inherent and integrative tendencies for CPD.

Factor 3 — Lacking the intention for CPD. It relates to the reduction in teachers’ motive to initiate or persist in goal-directed actions. Teachers are amotivated when they are in a state in which they are neither intrinsically nor extrinsically encouraged. In other words, they do not perceive a relationship between outcomes and their own behaviors (Ryan and Deci, 2002). In terms of SDT theory, this lacking of intention in CPD activities (Q26, Q31, Q21) is said to fall under the category of amotivation — a third type of motivational construct on the SDT motivation continuum. Specifically, amotivation comprises the nethermost extreme whilst intrinsic motivation holds the uppermost (Gagne and Deci, 2005). In a sense, the absence of motivation can lead to feelings of disintegration and encumber productivity (Ford and Roby, 2013). Fortunately, in this case of CPD motivation investigation, teachers’ response to these items has shown a positive tendency toward the relationship of CPD activities and their own actions. As for Q21, Q26, Q31, the percentages of respondents choosing “strongly disagree” and “disagree” are 69.06%, 67.08%, 70.55%, respectively, which indicates that teachers’ motivational profiles are characterized by low levels of amotivation.

Factor 4 — Academic and social responsibility. It consists of five items associated with teachers’ expectations to meet academic and social requirements. This factor highlights teachers’ behavior for the avoidance of guilt or anxiety and the attaining of ego-enhancements. This finding is buttressed by the “external expectations” in Boshier’s (1977) research and “compliance with authority” in confirming the measurement model underlying the “EPS-M” (Education Participation Scale-Modified) by Dia et al. (2016).

Attending CPD programs may not be optional in most higher education institutes in the Chinese context. Teachers performing such actions may feel pressured because of the school or department policy (Q17, Q25). Through the lens of SDT theory, this factor is related to introjected regulation, with which teachers begin to internalize their reasons for taking part in CPD activities. Put differently, teachers implementing the act because that’s what good teachers are supposed to do (Q24, Q27, Q28). This motive reflects teachers’ tendency to maintain and enhance their self-esteem or self-worth. However, in terms of SDT, this factor is not truly self-determined though teachers may feel that the pressure comes from within. It is partly internalized and considered still a form of controlled motivation.

Factor 5 — Academic self-fulfillment and obligation. It shows that teachers consciously endorse and identify with the value of participating in CPD activities. They perceived that CPD programs can be beneficial not only to teachers’ knowledge construction but also the pedagogical instruction and future academic advancement (Q2, Q1, Q3). Although this professional learning is possibly of no particular interest to teachers, they accepted it as a life goal, feeling obliged and responsible for it (Q4). This factor, in the eyes of SDT, is termed identified regulation. Different from factor 4, teachers with such a motivation experience a relatively higher degree of willingness to act. Even professional learning is not inherently enjoyable, teachers view it as worthwhile and meaningful.

Five types of TEFL teachers’ motivation for CPD have been identified in this study, which fall under the umbrella of SDT motivation continuum. In specific, these five factors appear to match amotivation, extrinsic motivation (extrinsic regulation, introjected regulation, identified regulation), and intrinsic motivation — the three major types of motivation along the SDT continuum proposed by Deci and Ryan (2000). Factor 1, factor 4, together with factor 5 are referred to as extrinsic motivation since teachers’ intention for CPD programs concentrates on separable outcomes, such as promotion, relationship, status, etc. These factors are in stark contrast to factor 2 — cognitive interest and inner-directed academic improvement, whose main concern is individual’s inherent satisfaction. Furthermore, it is also worth mentioning that factor 3 — lacking motivation for CPD, has been extracted in this research, which was an inseparable part of the motivation continuum and an important aspect to understand human nature.

### Levels of TEFL Teachers’ Motivation for Continuing Professional Development

The data analysis, with reference to the theoretically sound structure of SDT in this section, aims to address the second question of this study, that is, the extent to which teachers are intrinsically and extrinsically motivated for CPD. The result of EFA appeared to be situated along the SDT motivation continuum with amotivation (lacking the intention for CPD) anchored at the nethermost point whilst intrinsic motivation (cognitive interest and inner-directed academic improvement) the uppermost. The scores of items contained in the five dimensions were averaged to represent the TEFL teachers’ level of CPD motivation. Table 3 shows the distributive statistics of these respondents’ motivation toward continuing professional development.

**TABLE 3:** Distributive statistics for TEFL teachers’ CPD motivation.

Dimension	Minimum	Maximum	*M*	*SD*	Skewness	Kurtosis
Intrinsic motivation (inner-directed academic improvement and cognitive interest)	1.43	5.00	4.08	0.68	−0.54	0.38
Identified regulation (academic self-fulfillment and obligation)	1.00	5.00	4.28	0.67	−1.01	1.88
Introjected regulation (academic and social responsibility)	1.20	5.00	3.67	0.72	−0.29	0.15
External regulation (social recognition and promotion)	0.57	5.00	3.21	0.87	−0.12	−0.12
Amotivation (lacking the intention for CPD)	0.00	5.00	2.03	1.01	0.79	0.04

Table 3 demonstrates that of the five dimensions of CPD motivation, teachers’ identified regulation was at the highest level (*M* = 4.28), followed by intrinsic motivation (*M* = 4.08) and introjected regulation (*M* = 3.67). External regulation ranked at fourth place (*M* = 3.21), whereas amotivation was at the lowest level (*M* = 2.03). Overall, TEFL teachers were strongly motivated toward CPD activities, their identified regulation and intrinsic motivation were very strong. What is worth noting is that, although identified regulation falls under the category of extrinsic motivation, it is regarded as relatively autonomous. According to SDT theory, intrinsic motivation and identified regulation can be considered and understood as autonomous motivation (Deci and Ryan, 2000). Thus, the dominant motivation governing teachers’ CPD appeared to be less controlled by external factors. This could also be implied through the following table (Table 4) showing the frequency distribution of teachers’ response to each item. For the sake of clarity, this table only reports percentages of disagreement (strongly disagree 1 and disagree 2), neutral (score 3), agreement (agree 4 and strongly agree 5).

**TABLE 4:** Frequency of responses to CPD motivation (%).

Component	Simplified content in item	Agree	Neutral	Disagree
Intrinsic motivation — inner-directed academic improvement and cognitive interest	**18.** To satisfy my inquiring mind. **20.** To realize my self-worth. **19.** For the pursuit of high-level academic papers. **23.** To improve my academic level and knowledge. **15.** Like the feeling of constantly exploring and researching. **16.** To improve my critical thinking ability. **22.** To contribute to relevant academic field.	82.7 80.0 66.3 85.6 86.6 85.6 53.7	14.6 16.6 25.5 11.1 10.4 12.9 35.2	2.7 3.4 8.2 3.3 3 1.5 11.1
Identified regulation — academic self-fulfillment and obligation	**17.** My school supports them. **25**. To meet the requirements of performance evaluation in my school. **24**. To fulfill a kind of social responsibility. **27**. To keep up with competition. **28**. To supplement a narrow previous education.	61.6 51.7 57.4 64.9 75.5	28.7 29.5 28.2 25.3 17.8	9.7 18.8 14.4 9.8 6.7
Introjected regulation — academic and social responsibility	**2**. To seek knowledge for its own sake. **1**. To better guide my teaching. **3**. For my future academic development. **4**. It is teachers’ duty to participate in CPD programs.	84.2 85.2 86.9 76.2	12.6 11.1 10.9 16.6	3.2 3.7 2.2 7.2
External regulation — social recognition and promotion	**33**. To improve my social relationships. **34**. To maintain and improve my social status **32**. To be accepted by others. **8**. To gain recognition from my colleagues. **7**. To increase income. **14**. To catch up with others. **29**. To gain promotion.	29.5 34.2 25.8 51.5 53.2 45.8 51.2	35.2 33.4 35.2 33.7 32.4 33.2 35.4	35.3 32.4 39.0 14.8 14.4 21 13.4
Amotivation — lacking the intention for CPD	**26**. Do not see the relevance of going to CPD. **31**. Do not know the reasons of participating in CPD. **21**. Do not understand the purpose of going to CPD.	14.1 12.1 13.6	18.3 16.8 16.1	67.6 71.1 70.3

As can be seen in Table 4, items of intrinsic motivation were endorsed by large proportions of the respondents. For instance, over 80% of them confirmed their cognitive interest in learning (Q18, Q15, Q23). Items of identified regulation also received relatively adequate support from respondents with the percentages ranging from 51.7% to 75.5%. As the dominant motivational types in this study, these two motives demonstrated that the main driving force for TEFL teachers’ CPD activities was their pursuit of inherent enjoyment or meaningful outcomes. This was slightly different from Peng and Gao’s (2019) research on the relationship between research motivation and productivity, which uncovered the fact that academics mainly do research for external contingencies. In the current study, as can be seen from Table 4, the category of external regulation was embraced by just small proportions of the participants who admitted the externally imposed rewards. It is likely that Chinese academics are increasingly aware of the significance of CPD for their personal and professional advancement. They consciously identified and personally endorsed the value of participating in CPD activities, and they experienced a high degree of willingness and volition to take relevant actions. This was also supported by the percentages for disagreement in the dimension of amotivation. In a collective society such as China, individuals are more encouraged to do what is good for the community. Although the CPD programs, to some extent, are mandatory, teachers may be obligated to internalize the CPD tasks for the better performance of the school. This can be seen in the category of introjected motivation in Table 4 where items such as “It is teachers’ duty to participate in CPD programs,” “to better guide my teaching,” were affirmed by big proportions of respondents.

Chinese TEFL teachers tended to be less dependent on external incentives, but rather do CPD activities for inherent interest. Although extrinsic motivation (external regulation, introjected regulation, identified regulation included) in this study, still gained the upper hand, it implied a relatively different result, compared to the previous studies which underscored the influence of external regulation over intrinsic motivation (Bai and Hudson, 2011; Xie et al., 2014; Peng and Gao, 2019). In the current study, as can be seen from Tables 3, 4, teachers’ external regulation for CPD was weaker than their intrinsic motivation and their motivation for CPD showcased a higher degree of willingness and autonomy. The result echoed the findings of some empirical studies validating the dominance of language teachers’ intrinsic motivation over extrinsic motivation (Kassabgy et al., 2001; Erkaya, 2012).

As regards the differences between teachers of different demographic factors, the study conducted the *t*-test and one-way ANOVA in order to address the issue. A significant difference could be found in groups of male and female TEFL teachers. In specific, male teachers maintained a weaker motivation than their female counterparts in intrinsic motivation (sig. = 0.005, *t* = −2.84) and identified regulation (sig. = 0.002, *t* = −3.17) whilst in the dimension of amotivation, males were stronger than females (sig. = 0.003, *t* = 2.96). A possible explanation for this could be that men are faced with more financial and social pressure traditionally, but further empirical evidence is required.

And of the other demographic factors examined, the research found a significant difference in external regulation and introjected regulation amongst teachers of different years in teaching (see Table 5). Table 5 shows that both external regulation and introjected regulation were significantly varied by the years in teaching [*F*(3,398) = 9.06, *p* < 0.05; *F*(3,398) = 3.54, *p* < 0.05]. Bonferroni’s *post hoc* procedure indicated that, in the dimension of external regulation, those with less than 5 years of teaching experience were more externally motivated than their counterparts with 11–20 years of teaching and those with above 20 years of teaching experience. Moreover, there was also a significant difference between teachers with 5–10 years of teaching experience and those with above 20 years. The former was more driven by this type of motivation than the latter. Meanwhile, as far as introjected regulation was concerned, the *post hoc* statistics showed that teachers with less than 5 years of teaching experience were more motivated than those with 5–10 years of teaching experience and those of 11–20 years.

**TABLE 5:** Comparison of years in teaching on CPD motivation.

	(1) Less than 5 years (*n* = 89)		(2) 5–10 years (*n* = 68)		(3) 11–20 years (*n* = 149)		(4) Above 20 years (*n* = 96)		*F*(3,398)	*Post hoc* (Bonferroni)
	*M*	*SD*	*M*	*SD*	*M*	*SD*	*M*	*SD*		
External regulation	3.57	0.80	3.30	0.75	3.19	0.87	2.92	0.90	9.063*	(1) > (3) (1) > (4) (2) > (4) (3) > (4)
Introjected regulation	3.89	0.68	3.57	0.66	3.62	0.76	3.64	0.70	3.540*	(1) > (2) (1) > (3)

*p < 0.05.

This was somewhat different from the study conducted by Zhang et al. (2021) in which the statistics showed a significant association between teachers’ teaching experience and autonomous motivation. It was stated that teachers with more teaching experience were less autonomously motivated for professional learning activities. However, in the current case, it seemed that the more teaching experience teachers have, the less extrinsically motivated they are to take part in those CPD activities. The main reason for this discrepancy possibly lies in the programs designated to the research. In the study of Zhang et al. (2021), the professional program only referred to a specific educational program refining teachers’ pedagogical strategies. Teachers with more teaching experience may be less in favor of interactions about their subject domain. Nevertheless, in the current study, teachers with less teaching experience, for instance, the new teachers, were usually under the heavy pressure of performance evaluation. So they tended to associate professional development more with external contingencies and considered it a separable task. Besides, their innate psychological needs, namely, competence, autonomy, and relatedness, were relatively far away from being satisfied, which, according to SDT (Ryan and Deci, 2000b), may reduce their intrinsic motivation.

In addition to the difference analysis, the correlation result found no correlated relationship among varied variables. Overall, research data found that the most dominant motivation for TEFL teachers’ CPD was the identified regulation which was followed by intrinsic motivation. These two types of motivation revealed that teachers’ behavior for CPD were characterized by a relatively high degree of autonomy. In other words, TEFL teachers’ actions for CPD started to become more congruent with their personal objectives and identities. The findings were somewhat different from the motivation studies conducted previously (Xie et al., 2014; Peng and Gao, 2019; Liu et al., 2020), which underscored the influence of external regulation over intrinsic motivation. Of the demographic factors analyzed, there was a significant difference between teachers of different gender and different years of teaching experience.

### Struggles and Its Implications in Sustaining Teachers’ Continuing Professional Development Motivation

Although the above research data indicated that Chinese TEFL teachers’ motivation for CPD is strong in general, problems and challenges remained regarding the sustaining and enhancing of teachers’ CPD motivation. Analyses of the qualitative data — the open-ended item (Q35) asking about respondents’ difficulties in CPD and the followed-up interviews with 12 TEFL teachers, revealed several themes, and this section presented the emergent themes in addressing the third research question.

This study designed a specific open question asking about the difficulties and challenges teachers encountered in CPD. Since this question placed at the end of the survey was optional, 141 answers were received out of 402 questionnaires. Moreover, to better understand the opinions stated in the open-ended item, the followed-up interviews with 12 TEFL teachers were conducted. The responses from the last item and the transcription of the interviews were then processed by content analysis. A qualitative analysis software — Nvivo (Version 12) was adopted to assist the data coding. The procedures and details as regards the codification of the qualitative data has been presented in the part of “Data analysis” in this paper (see the section “Data Analysis”). This section mainly reports the findings of the content analysis.

Table 1 presents the three main categories of difficulties TEFL teachers faced in terms of CPD. These factors inhibiting teachers’ motive for CPD are school-related problems, CPD-related problems, and teacher-related problems. These dimensions confirm the model proposed by McMillan et al. (2016). In their model, they offered a comprehensive view of factors influencing teachers’ motivation for CPD at three levels: personal level, school-related level, and system-wide level.

In the current study, at the school level, limited financial support and few opportunities afforded by universities were frequently referred to as causes impeding teachers’ professional development. This mainly concerned the general support in school. It is assumed that supportive school culture is positively linked to both autonomous motivation and controlled forms of teachers’ motivation for professional learning (Zhang et al., 2021). However, in the current case, teachers’ responses indicated that the support from school policy was not that strong. This was especially true for teachers from ordinary universities. Teacher 1 explained this in her interview:

*“Although my university is quite supportive of teachers’ CPD, as a school in the central part of the country, the funding for CPD is not that sufficient. So not all academics have the chance to go to the foreign language forums or other summer forums. Teachers with higher academic title or with administrative title get more involved in these activities, because they need to be acquainted with the academic focus and trends in the field.” (Teacher 1, Professor of English linguistics).*

Factors at the school level are closely linked to the effectiveness of teachers’ professional learning experience (Zhang et al., 2021). To trigger the intrinsic and extrinsic motivation of teachers, social conditions are required to play the supportive role.

As regards the second category, that is, the problems associated with CPD programs *per se*, issues like limited types of CPD, in particular, “lack of ESP (English for Special Purpose) -related CPD programs” and the imbalance between theory and practice did concern them. Teacher 4 in the interview expressed her anxiety toward the CPD programs:

*“Colleges and universities continue to reduce college English hours and credits. I understand that it is an inevitable trend. But I felt that there are not enough training opportunities for me to appropriately deal with issues as regards teaching ESP. I would like to improve myself in this field. ESP-related. I am looking forward to taking part in more CPD projects that concentrate on ESP teaching.” (Teacher 4, Associate professor of English linguistics).*

Teachers’ struggles in this dimension roughly corresponded to the system-wide motivation factors in the research of McMillan et al. (2016). This category was mainly in relation to teachers’ engagement in the CPD activities. Teachers were provided mandatory courses during school hours, and the courses offered were irrelevant to teachers’ field of study. Furthermore, a frequently mentioned factor concerned the imbalance between theory and practice. This was echoed by respondents who were eager to make improvements in terms of ESP-teaching. The imposition of compulsory CPD programs may automatically preclude teachers’ intrinsic motivation. Therefore, it could be said that these CPD system-wide problems further attested to the fact that social conditions make a big difference to teachers’ motivation. According to SDT, social conditions can either be supportive of or impede individuals’ development, depending on the role the conditions play in meeting individuals’ psychological needs (Power and Goodnough, 2018). When conditions are favorable to these needs, individuals’ motivation can be fostered, but if conditions thwart the need fulfillment, individuals’ motivation and development are to be curtailed (Deci and Ryan, 2008a).

At the personal level, that is, the teacher-related problems: teachers’ autonomy, relatedness and competence, were labeled as factors stimulating or inhibiting teachers’ CPD motivation. In the eyes of SDT theory, autonomy means that people take actions in a volitional manner and they perceive themselves as the cause or source of their behavior; relatedness refers to individual’s feeling of being connected to others in satisfying and supportive relationships; while competence is rather a reflection of a person’s sense of efficacy and confidence (Deci and Ryan, 2008a).

As can be seen in Table 6, competence (31 references) was the factor most frequently referred to as the challenge teachers encountered in CPD. Geijsel et al. (2009) in their study stated that teachers who were confident about their own capabilities showed more enthusiasm about learning and were more engaged in learning activities. This positive relationship could also be found between teacher self-efficacy and autonomous motivation (Zhang et al., 2021). In addition to the factor of teachers’ competence, teachers’ autonomy and relatedness were also labeled as factors influencing teachers’ CPD activities. But it is worth noting that these two psychological needs possessed fewer references compared to the factor of competence. Specifically, there were nine references for autonomy and three references for relatedness. This could imply that autonomy and relatedness were less associated with teachers’ motivation for CPD. In fact, a recent study conducted in China, identified that colleague support and task autonomy were unrelated to Chinese teachers’ motivation for professional development (Zhang et al., 2021), which contradicted some of the findings in the Western context highlighting the significant positive relationship between colleague support, task autonomy and teachers’ motivation for professional learning (Kwakman, 2003; Thoonen et al., 2011). This contrast may be attributed to cultural differences. In Eastern culture, people value community over the individual, and it is selfish to cause someone to lose face whilst in Western culture, people attach more importance to their own needs (Nhung, 2014).

**TABLE 6:** Difficulties and challenges teachers encountered in CPD.

Themes (percentage)	Sub-themes (reference)	Examples of initial concept
School-related problems (32.6%)	Inadequate funding (15)	There is no CPD funding for us; The training costs me a lot; I can’t afford it; A large proportion of the CPD cost is at my own expense; The school provides little reimbursement.
	Few spots (18)	“There are too many (Buddhist) monks and too little gruel”; Places for CPD are very limited; Not much opportunities for us to go out for training; Few individualized CPD opportunities.
CPD-related problems (24.8%)	Few types of CPD (15)	Lack of ESP (English for Special Purpose) -related CPD programs; Lots of CPD programs are just not for your field of study; CPD programs should cater for teachers’ professional needs; The current CPD programs are homogenized; Lack of individualized CPD projects.
	Emphasize theory over practice (10)	There is too much theoretical knowledge; The gap between the theory learned in CPD and the teaching practice remains huge; They should provide more practical programs guiding the transform from teaching EGP (English for General purpose) to ESP (English for Special Purpose).
Teacher-related problems (basic psychological needs) (42.6%)	Autonomy (9)	It is irrelevant with my field, and I gained a little; CPD programs clash with my own class; There is few CPD options.
	Competence (31)	I’m poor in theoretical foundation; It’s hard for me to gain proficiency in computer skills; unable to keep pace with the academic trends; My learning in CPD is really fragmented, not systematic; It is challenging to implement ESP-oriented curriculum transform.
	Relatedness (3)	It’s difficult to find someone who has the same academic interest with me; I couldn’t find a team to study with.

Overall, compared to autonomy and relatedness, TEFL teachers’ psychological need for competence stood out as a major factor impacting teachers’ involvement in CPD. From the interview with teacher 6, we could have a concrete example of this problem.

*“A lot of research these days is about mining data, analyzing statistics or construct models. But to be honest, it’s a big challenge for people with the background of liberal arts. Although I participated in some continuing education activities, I could hardly keep pace with them. I was upset and discouraged” (Teacher 6, Associate professor of English).*

Furthermore, among the examples of the theme — teacher-related problems, the shift to teaching ESP from EGP had also been referred to by most interviewees. For example, Teacher 11 mentioned in his interview *“The aim of college English teaching should not just concentrate on training basic English language skills. Instead, it should help students acquire and exchange academic information”* (Teacher 11, lecturer of English literature). Teachers’ recognization of curriculum reform from EGP to ESP prompted them to take the initiative to make adaptations. However, although they recognized the significance of EGP in satisfying students’ needs in studying abroad, paper publication, professional study and other aspects of personalized demand, they were concerned about their capability of implementing the plan. In other words, though this “identified regulation” has shown a high degree of autonomy in teachers’ behavior, they are still not intrinsically motivated. what should also be noted is that different types of psychological needs are assumed to be equally significant for healthy human functioning (Janke et al., 2015). Hence, according to SDT, more should be done to deal with teachers’ three psychological needs in order to internalize their external motivation.

### Implications

The analysis of teachers’ struggles in the three categories in Table 6 indicated that teachers’ motivation for CPD could be fostered and triggered in terms of the following aspects under the framework of SDT theory. Firstly, financial support from governments and universities should be rendered to TEFL teachers continuously. Although the past decades witnessed a steady increase in the investment of teachers’ CPD, for instance, the support from the Ministry of Education and the Chinese Scholarship Council for teachers to make academic visits to universities home and abroad (Peng and Gao, 2019), the needs for various forms of CPD programs are far from being addressed. Secondly, the CPD practices could attach great importance to the specific needs of learners. In other words, more individualized and teaching practice-oriented programs, instead of externally driven, one-off ones, should be promoted. These conditions, in the view of SDT theory, are said to be social factors that could contribute to the fostering of intrinsic motivation or autonomous motivation. The benefits of intrinsic motivation within formal education are proved by a plethora of empirical studies (Ryan and Deci, 2020). Thus, at the micro-level, teachers’ inherent interest in CPD needs to be triggered. This leads to the suggestion that TEFL teachers obtain direction concerning the specific solutions toward their basic psychological needs of “competence,” “autonomy,” and “relatedness.” For example, colleges and universities should provide teachers cognitive, material and institutional support for them to participate in the reform (EGP-ESP) -related CPD programs. Specifically, more efforts should be made in terms of the following two respects.

First, to emphasize on normalizing teachers’ continuing professional development. Teachers’ job satisfaction, teaching motivation, research motivation and motivation for professional development are susceptible to both internal and external factors. This could be coped with by attaching importance to the dynamics of teacher motivation and the application of “community of practice” (Lave and Wenger, 1998). The formation of “community of practice” is based on teachers’ similar interest in learning, research and professional development. The practice promotes orderly growth and development among teachers of community. In-service college English teachers could share their gains, ask for help for their frustrations, and delve into the problems they encountered in the teaching practice with other members in the team. Hence, the effect of the instability of teacher motivation for CPD could be countered.

Second, to explore the diverse mode of teacher CPD education and promote the concept of life-long learning. As was mentioned in participants’ interviews, the duration of COVID-19 has thwarted their attempts in engaging in traditional professional development programs. Teachers were asked to opt for online approaches in terms of CPD education. The shift from traditional mode to non-traditional mode is bound to bring about innovation in both educational concept and methods. Hence, as far as teachers’ continuing education is concerned, multiple modes rather than single forms are advocated for a better development of teacher profession. Besides, in a society embracing life-long learning, continuing education has a responsibility to promote language teachers to be practitioners of this philosophy. Teachers adhering to the idea of life-long learning are more likely to be intrinsically motivated to fulfill their potentials in education.

To sum up, research data (Tables 3, 4) demonstrated that TEFL teachers achieved relatively strong intrinsic motivation for CPD in this study. To maintain and enhance the intrinsic motivation, social support was expected to be more relevant and effective in meeting teachers’ basic psychological demands. The corresponding measures above have a prospect in promoting teachers’ full engagement in the professional learning process and facilitating their optimal growth.

## Conclusion

With TEFL teachers in the Chinese context undergoing important transitions and reforms in respect of the teaching goals, curriculums goals, curriculums, and standards, TEFL teacher education is facing both challenges and opportunities for further development. Although substantial progress has been made in this respect in the past decades, much more needs to be done to ensure its relevance and effectiveness. The research into the motivating and inhibiting factors as regards teachers’ engagement in CPD would provide valuable insights for the strengthening of teacher education. The current study examined this issue through mixed research methods under the framework of SDT theory. The quantitative analysis found that five motivational orientations, namely, inner-directed academic improvement and cognitive interest, academic self-fulfillment and obligation, academic and social responsibility, social recognition and promotion, lacking the intention for CPD, emerged as the TEFL teachers’ main tendencies engaging in CPD. TEFL teachers, in general, were positively motivated to participate in CPD activities. The results also shared some differences with previous studies in that the participants exhibited stronger identified regulation and intrinsic motivation than external regulation, which was slightly inconsistent with the prior findings highlighting external regulation. For the demographic factors examined, this study found a significant difference in external regulation and introjected regulation amongst teachers of different gender and of different years in teaching. The qualitative discussion suggested that support from social conditions should help to facilitate the effective functioning of teachers’ intrinsic or autonomous motivation and bring desirable consequences to CPD.

Although this study attempted to provide a comprehensive focus on TEFL teachers’ motivation for continuing professional development, it is not exempt from limitations. The sizes of different groups, such as gender, affiliation, were uneven. It would be desirable that the groups were reasonably well represented to enrich the findings of CPD motivation. Moreover, this research, being descriptive and explanatory in nature, just concentrated on the types and levels of teachers’ CPD motivation. It is suggested that future study endeavor to explore the relationships between teachers’ CPD motivation and teacher outcomes as well as students’ learning outcomes for validating the theoretical framework. In addition, it is advised that more research methods, for instance, ethnographic or narrative approaches, should be employed in order to better understand the internalization of individual teachers’ CPD motivation in the future. Besides, future studies can also embark on more qualitative data to provide a better picture of what TEFL teachers deem indispensable for their professional development. One new line of inquiry could be the impact of teacher-student interpersonal variables that can have a bearing on nurturing and nourishing teachers’ and students’ success, growth, and well-being (Xie and Derakhshan, 2021).

## Data Availability Statement

The raw data supporting the conclusions of this article will be made available by the authors, without undue reservation.

## Ethics Statement

The studies involving human participants were reviewed and approved by Henan University Academic and Ethics Committee. The patients/participants provided their written informed consent to participate in this study.

## Author Contributions

JY contributed to the conceptualization, methodology design, data collection, writing – original draft, investigation, software, validation, and writing.

## Conflict of Interest

The author declares that the research was conducted in the absence of any commercial or financial relationships that could be construed as a potential conflict of interest.

## Publisher’s Note

All claims expressed in this article are solely those of the authors and do not necessarily represent those of their affiliated organizations, or those of the publisher, the editors and the reviewers. Any product that may be evaluated in this article, or claim that may be made by its manufacturer, is not guaranteed or endorsed by the publisher.

## Footnotes

1
www.wjx.cn
